# Ambivalence among intergenerational relationships in old age

**DOI:** 10.1192/j.eurpsy.2021.1970

**Published:** 2021-08-13

**Authors:** S. Von Humboldt, A. Costa, S. Silva

**Affiliations:** 1 William James Center For Research, ISPA-Instituto Universitário, Lisbon, Portugal; 2 Ispa – Instituto Universitário, ISPA – Instituto Universitário, Lisbon, Portugal

**Keywords:** older adults, ambivalence, Adult children, intergenerational relationships

## Abstract

**Introduction:**

This study focuses on ambivalence among intergenerational relationships in old age.

**Objectives:**

This study aims to analyze the perspectives of intergenerational relationships between older adults and adult children. For this purpose, a qualitative research was carried out, which analyzes these relations at a cross-national level.

**Methods:**

Four hundred and twenty four older participants aged 65-97 years, were interviewed. Participants were of three different nationalities and lived in the community. All the interviews went through the process of verbatim transcription and subsequent content analysis.

**Results:**

Two dimensions of generational ambivalence were revealed from the study; support and the conflict dimensions. Findings of content analysis produced six themes, which represent intergenerational relations between older adults and adult children: older adults-adult children interaction quality; family integration; care and support; definition of limits; distance and alienation; and communication difficulties.

**Conclusions:**

This study highlighted the diversity of experiences in old age, in relation to intergenerational relationships and underlined the conflicting expectations from older adults in relation to their adult children.

**Disclosure:**

No significant relationships.

